# Clinical judgment is the most important element in overhydration assessment of chronic hemodialysis patients

**DOI:** 10.1007/s10157-012-0745-9

**Published:** 2012-11-29

**Authors:** Radovan Vasko, Gerhard A. Müller, Brian B. Ratliff, Klaus Jung, Sarah Gauczinski, Michael J. Koziolek

**Affiliations:** 1 Department of Nephrology and Rheumatology, Georg-August-University Göttingen, Robert-Koch-Str. 40, 37075 Göttingen, Germany; 2 Department of Medical Statistics, Georg-August-University Göttingen, Göttingen, Germany; 3 Department of Medicine, New York Medical College, New York, USA

**Keywords:** Hemodialysis, Overhydration, Clinical judgment, Bioimpedance

## Abstract

**Background:**

The assessment of hydration status remains a challenging task in hemodialysis (HD) management. There are only limited data available on the relevance of clinical decisions in the estimation of dialysis overhydration (OH). The objective of this study was to examine the significance of clinical judgment in the assessment of pre-dialysis OH.

**Methods:**

We compared the performance of three methods of OH assessment: (1) clinical judgment guided by a single clinical examination with (2) multifrequency bioimpedance analysis (BIA) and (3) complex systematic clinical approach. We additionally studied the associations of these methods with selected laboratory and imaging parameters.

**Results:**

Any of the single parameters alone reached a sufficient level of accuracy for reliable prediction of OH. Clinical judgment was the single most important factor in OH estimation, and also had the highest contribution when in combination with other parameters. BIA reliably measured extracellular fluid, but the automatically calculated OH_BIA_ exhibited a substantial degree of inaccuracy that precludes the use of BIA as a standard at present. The combination of clinical judgment with additional clinical parameters had the highest prediction accuracy for OH. Among the parameters studied, vena cava collapsibility index and calf circumference showed the strongest association with OH. Echocardiography, cardiothoracic index, atrial natriuretic peptide levels and spirometry did not have acceptable sensitivity.

**Conclusion:**

The systematic clinical approach combining physician and patient inputs, laboratory and imaging data enables an individualized decision and a superior accuracy in OH assessment.

**Electronic supplementary material:**

The online version of this article (doi:10.1007/s10157-012-0745-9) contains supplementary material, which is available to authorized users.

## Introduction

While the assessment of solute clearance has moved forward substantially in recent years, the estimation of adequate fluid removal remains a challenging problem in the management of hemodialysis (HD) patients. Dialysis-associated overhydration (OH) and dehydration have been linked to adverse events. Chronic OH is a major factor in the development of arterial hypertension, although the causal relationship between OH, hypertension and mortality is intricate due to the higher prevalence of comorbid conditions in HD patients. Hypotension resulting from excessive ultrafiltration can provoke acute ischemic events with recurrent episodes, potentially causing functional impairment and organ damage [1, 2].

Dry weight (DW) has been conventionally defined as the lowest weight that can be tolerated without developing symptoms of hypovolemia. Although based on trial and error, probing for DW has been a common practice. Today, it is not simply a symptom-guided probing anymore, but rather a complex systematic clinical approach, including laboratory data and imaging techniques. Patient-reported symptoms can be misleading without knowing the medical history and usually become more specific as the OH increases [3]. Patients differ in autonomic system responsiveness, vascular refilling capacity, comorbidities and their therapy. Advanced kidney disease is accompanied by metabolic alterations, often resulting in decrease in body cell mass, increase in extracellular volume and consequently OH. Body composition undergoes changes yet again after a patient starts HD treatment and the uremic environment improves. All this together makes an accurate assessment of hydration in HD patients very challenging. The role of many laboratory and imaging parameters as potential indicators of OH has been evaluated, but with inconsistent results. Bioelectrical impedance (bioimpedance, BIA) offers the possibility of direct measurement of extracellular and intracellular fluid compartments [4]. It gained more attention in recent years when several studies reported superiority of BIA in the assessment of dialysis OH [5]. Unfortunately, with the introduction of new technologies, there has been an indisputable tendency to undervalue the significance of clinical judgment in hydration status estimation.

The objective of the present study was to evaluate the relevance of clinical judgment in the assessment of pre-HD OH. To accomplish this, we compared the performance of three different methods of OH estimation: (1) clinical judgment guided by a single clinical examination with (2) multifrequency bioimpedance analysis and (3) complex systematic clinical approach. We additionally examined the associations of these methods with selected laboratory and imaging parameters.

## Subjects and methods

### Patients

Thirty patients with end-stage renal disease receiving HD were enrolled in the study. They did not have any acute illness and their DW was stable in the previous 3 months. Subjects were not included if one or more of the following were present: younger than 18 years of age, implantable electronic medical devices, metal artificial joints or limb amputation. HD was performed three times per week using a low-flux polysulfone dialyzer and a Fresenius F4008 HD machine. The study protocol was approved by the local ethics committee and informed consent was obtained from all subjects.

### Measurements

Age, gender, body weight and height were documented and blood samples obtained from each patient. Reference overhydration (OH_REF_), used as a standard, was calculated as the difference between pre-HD weight and DW. DW was determined by the managing physicians (dialysis physicians not participating in the study) using the long-term (weeks to months) systematic clinical approach including patient history, symptoms, laboratory parameters and routine diagnostic techniques (echocardiography, ultrasonography, chest X-ray), but not BIA. Clinical overhydration (OH_CLI_) represents the clinical judgment of two nephrologists (not involved in the treatment of study patients), which estimated OH_CLI_ guided by single clinical examination, patients’ history and symptoms. They were not aware of patients’ DW and laboratory parameters. Blood pressure (BP) was recorded as a mean of three consecutive pre-HD readings. Echocardiography was performed with a Philips Sonos 5500, and vena cava diameter (VCD) was measured with an Esaote Technos MPX system, both before HD. Vena cava collapsibility index (VCCI) was calculated as (VCDexp − VCDinsp)/VCDexp. The lower the VCCI, the higher the likelihood that patient is volume-overloaded. The cardiothoracic index (CTI) was calculated by dividing the horizontal width of the cardiac silhouette by the maximal transverse thoracic diameter on a chest radiograph. Vital capacity was measured in a standing position before HD.

### Bioimpedance

Multifrequency bioimpedance analysis (BIA) was performed using a Hydra 4200 system (Xitron Technologies, San Diego, CA, USA). Extracellular (ECW), intracellular (ICW) and total body water (TBW) were measured. Bioimpedance overhydration (OH_BIA_) was calculated automatically by the integrated fluid management software (Version 1.22, Fresenius Medical Care). Measurements were performed at the bedside, in standardized conditions as previously described [6]. During the measurement, patients were not allowed to drink or eat. The first electrode pair was placed on the dorsal surface of the wrist and on the dorsal surface of the third metacarpal bone. The second pair of electrodes was positioned on the anterior surface of the ankle and on the third metatarsal bone. All measurements were taken by the same operator. Intraobserver variability was analyzed by repeated measurements in a group of 13 patients, and was under 5 %.

### Statistical analysis

Statistical analyses were performed using SPSS 17.0 for Windows (SPSS, Chicago, USA). Correlations of parameters with OH were studied by Pearson’s correlation coefficient *R*. Parameters significant in the univariate analyses were combined in multiple regression models. Data are presented as mean ± standard deviation. *P* < 0.05 was considered statistically significant.

## Results

### Patients and demographics

The demographic and clinical characteristics of the patients are presented in Table 1. Mean age was 67 ± 12 years, with 60 % males and 33 % diabetics. The average length on dialysis was 3.6 years. The most common etiologies of ESRD were diabetic-hypertensive nephropathy and glomerulonephritis.

**Table 1:** Demographic and clinical characteristics of the patients

Variable	
Patients (male/female) (*n*)	30 (18/12)
Age (years)	67 ± 12 (46–85)
Diabetes (*n*)	10
HD vintage (years)	3.6 ± 2.5
Predialysis SBP/DBP/MAP (mmHg)	125 ± 18/71 ± 10/89 ± 11
Postdialysis SBP/DBP/MAP (mmHg)	110 ± 19/62 ± 11/78 ± 12
Height (cm)	167.9 ± 6.8
Dry weight (kg)	71.8 ± 14.4
OH_REF_ (kg)	2.6 ± 1.3 (0.9–5.6)
OH_CLI_ (kg)	2.4 ± 1.0 (1.0–5.0)
OH_BIA_ (kg)	3.6 ± 2.0 (−1.2–8.0)
TBW (L)	33.8 ± 8.8
ECW (L)	17.2 ± 3.7
ICW (L)	16.1 ± 5.1

*HD* hemodialysis, *SBP* systolic blood pressure, *DBP* diastolic blood pressure, *MAP* mean arterial blood pressure, *OH*_*REF*_ reference overhydration, *OH*_*CLI*_ clinically assessed overhydration, *OH*_*BIA*_ bioimpedance calculated overhydration, *TBW* total body water, *ECW* extracellular water, *ICW* intracellular water

### Overhydration

Pre-HD overhydration assessed by the systematic clinical approach (OH_REF_) was 2.6 ± 1.3 L, estimated by nephrologists (OH_CLI_) 2.4 ± 1.0 L and calculated by BIA (OH_BIA_) 3.6 ± 2.0 L. OH_CLI_ (*R* = 0.61, *P* < 0.001), but not OH_BIA_ (Table 2), correlated with reference OH_REF_. Since BIA directly measures ECW and calculates OH_BIA_, we substituted OH_BIA_ with ECW/BSA, and were able to show a correlation with OH_REF_ (*R* = 0.52, *P* = 0.01).

**Table 2:** Correlations between overhydration and selected biochemical and clinical parameters

Variable	OH_REF_	OH_CLI_	ECW/BSA
Age	−0.43 (*P* = 0.018)	NS	NS
OH_CLI_	0.61 (*P* < 0.001)		0.42 (*P* = 0.002)
OH_REF_		0.61 (*P* < 0.001)	0.50 (*P* = 0.005)
OH_BIA_	NS	NS	0.50 (*P* = 0.005)
Height	NS	NS	0.43 (*P* = 0.018)
Pre-HD weight	0.43 (*P* = 0.017)	0.55 (*P* = 0.002)	0.62 (*P* < 0.001)
Pre-HD SBP	NS	NS	NS
Pre-HD DBP	0.54 (*P* = 0.002)	NS	0.51 (*P* = 0.004)
Pre-HD MAP	0.38 (*P* = 0.042)	NS	0.48 (*P* = 0.008)
NT-proANP	NS	NS	NS
LAD, LVEDD	NS	NS	NS
VCCI	−0.45 (*P* = 0.013)	NS	−0.36 (*P* = 0.048)
Cardiothoracic index	NS	NS	NS
Vital capacity	NS	NS	NS
Pre-HD calf circumference	0.37 (*P* = 0.041)	0.47 (*P* = 0.009)	0.56 (*P* = 0.001)
ECW	0.52 (*P* = 0.003)	0.50 (*P* = 0.005)	0.94 (*P* < 0.001)

*OH*_*REF*_ reference overhydration, *OH*_*CLI*_ clinically assessed overhydration, *OH*_*BIA*_ bioimpedance calculated overhydration, *HD* hemodialysis, *SBP* systolic blood pressure, *DBP* diastolic blood pressure, *MAP* mean arterial blood pressure, *NT-proANP* N-terminal atrial natriuretic peptide, *LAD* left atrial diameter, *LVEDD* left ventricular end diastolic diameter, *VCCI* vena cava collapsibility index, *ECW* extracellular water, *NS* not significant

Pre-HD calf circumference was positively correlated with OH_REF_ (*R* = 0.37; *P* = 0.041), OH_CLI_ (*R* = 0.47; *P* = 0.009), ECW/BSA (*R* = 0.56; *P* = 0.001) and pre-HD weight (*R* = 0.68; *P* < 0.001), indicating a strong association of this simple anthropometric measure with fluid overload.

VCCI was negatively correlated with OH_REF_ (*R* = −0.45; *P* = 0.013), concordant with decreasing collapsibility of the vena cava with increasing OH.

In terms of fluid overload, N-terminal atrial natriuretic peptide (NT-proANP) levels did not correlate with OH or left atrial diameter (LAD) (although positively with CTI, *R* = 0.67; *P* < 0.001), parameters with known association in the normal population. The significant influence of impaired renal function on NT-proANP levels is evident by its positive correlation with serum creatinine levels (*R* = 0.57; *P* = 0.001).

### Blood pressure

The average pre-HD BP was 125/71 mmHg, post-HD BP 110/62 mmHg, and the average BP reduction was 6/3 mmHg per one liter of OH removed. The mean 24-h ambulatory BP on a HD-free day was 116/68 mmHg.

Pre-HD diastolic blood pressure (DBP) (*R* = 0.54; *P* = 0.002), mean arterial blood pressure (MAP) (*R* = 0.38; *P* = 0.04), but not systolic blood pressure (SBP) (*R* = 0.09; *P* = 0.64) correlated with OH_REF_. Interestingly, after HD, SBP (*R* = 0.43; *P* = 0.02), DBP (*R* = 0.37; *P* = 0.04) and MAP (*R* = 0.43; *P* = 0.02) were positively correlated with the number of antihypertensive drugs (no correlation was seen before HD).

Prognostic data are presented in the Electronic Supplementary Material.

### Multiple regression models

Parameters significant in the univariate analyses (Table 2) were combined in multiple regression models (Table 3). Calf circumference was considered as a part of the clinical examination and not explored separately. All models were characterized by sample-size-corrected coefficient of determination *R*² and Akaike’s information criterion (AIC). *R*² shows how good is the model in predicting the reference OH_REF_. AIC provides a means for comparing the goodness-of-fit of different models. The higher the *R*² and the lower the AIC, the better the model. As demonstrated in Model 1, calculated OH_BIA_ accounted for only 3 % of OH_REF_. However, after replacement with ECW/BSA, the prediction accuracy for OH_REF_ increased to 22 % (Model 2). From all single variables, the OH_CLI_ was most consistent and accounted for approximately 35 % of OH_REF_ (Model 3). The combination of several clinical parameters (age, pre-HD weight, pre-HD MAP, pre-HD DBP, and VCCI) had an accuracy of 51 % (Model 4). While the addition of ECW/BSA to Model 4 did not improve (49 %, Model 5) and ICW/BSA slightly improved (55 %, Model 6) the accuracy, the addition of OH_CLI_ significantly increased the overall precision (64 %, Model 7). In combination with clinical parameters and OH_CLI_, ICW/BSA (Model 9, predictor importance 0.11) is superior to ECW/BSA (Model 8, predictor importance 0.01).

**Table 3:** Overview of different models for estimation of reference overhydration (OH_REF_)

Model	Adj. *R*²	AIC	Variables	Predictor importance
1. OH_BIA_	0.03	16.5	OH_BIA_	1.0
2. ECW/BSA	0.22	8.0	ECW/BSA	1.0
3. OH_CLI_	0.35	2.7	OH_CLI_	1.0
4. Parameters	0.51	1.0	Age	0.11
			Pre-HD weight	0.21
			Pre-HD MAP	0.09
			Pre-HD DBP	0.19
			VCCI	0.39
5. Parameters + ECW/BSA	0.49	4.3	Age	0.13
			Pre-HD weight	0.13
			Pre-HD MAP	0.11
			Pre-HD DBP	0.22
			VCCI	0.40
			ECW/BSA	0.01
6. Parameters + ICW/BSA	0.55	−0.6	Age	0.09
			Pre-HD weight	0.23
			Pre-HD MAP	0.11
			Pre-HD DBP	0.21
			VCCI	0.25
			ICW/BSA	0.11
7. Parameters + OH_CLI_	0.64	−5.9	Age	0.19
			Pre-HD weight	0.01
			Pre-HD MAP	0.07
			Pre-HD DBP	0.13
			VCCI	0.24
			OH_CLI_	0.36
8. Parameters + OH_CLI_ + ECW/BSA	0.62	−2.5	Age	0.20
			Pre-HD weight	0.00
			Pre-HD MAP	0.08
			Pre-HD DBP	0.12
			VCCI	0.20
			OH_CLI_	0.39
			ECW/BSA	0.01
9. Parameters + OH_CLI_ + ICW/BSA	0.70	−8.7	Age	0.15
			Pre-HD weight	0.07
			Pre-HD MAP	0.10
			Pre-HD DBP	0.17
			VCCI	0.18
			OH_CLI_	0.22
			ICW/BSA	0.11

*AIC* Akaike’s information criterion, other abbreviations as in Table 2

## Discussion

An optimal method should have high sensitivity and specificity, while still being generally applicable and cost-effective. The systematic clinical approach is a system combining physician and patient inputs, laboratory data and imaging. Clinical judgment guided by clinical examination is a crucial component of the systematic clinical approach. Our models have identified clinical judgment as the single most important factor in OH assessment. BIA reliably measures ECW and calculates OH_BIA_ using a body composition model, based on reference data obtained from the normal population. Dry weight determined from the computerized OH_BIA_ cannot be always applied and achieved without the risk of dehydration and, therefore, does not represent the optimal DW in every patient. The discrepancy between measured ECW and calculated OH_BIA_ can be at least partly explained by not implementing the option for patient-individualized and/or disease-specific correction. Hence, the general applicability of the computed OH_BIA_ remains uncertain. We see a particular strength of BIA in the direct estimation of ICW that defines body cell mass and cannot be reliably assessed by other routine techniques. Malnutrition, a commonly undiagnosed condition in dialysis patients, leads to loss of lean body substance [7]. Implementation of serial ICW measurements in individual patients would be able to unmask a clinically inapparent decline in body mass, prevent an increase of OH, and uncover an underlying process possibly requiring further medical intervention. This interpretation is supported by our models, which selected ICW as the most significant BIA parameter in OH assessment. Our analyses make evident that only combinations of several methods and parameters provide an acceptable prediction precision. The integrative function of clinical judgment is reflected by the better accuracy of models with implementation of OH_CLI_ and also by the highest predictive importance of OH_CLI_.

Despite similar hydration characteristics, our patients had lower BP than the study subjects reported by Chazot [8]. However, many studies do not report antihypertensive drugs prescribed only for cardioprotection, which creates inconsistency. We think that this different indication does not eliminate the antihypertensive effect, and included them in our analysis. Investigators from Tassin in France described patients who remain normotensive despite being above calculated DW, and explain this by better clearance of vasoactive substances during the long HD practiced in the Tassin dialysis center [9]. Our patients presented a normal average BP that correlated with OH. This emphasizes that BP changes rather than absolute values in individual patients, even within normal limits, may be indicative of OH. Undetected overhydration, silent hypervolemia, may result in hypertension as late as 12 h after leaving HD [10]. For this reason, we believe that regularly performed 24-h BP monitoring should be a standard component of hydration evaluation in HD patients.

The calf has a relatively uniform structure with better hydration, and recent evidence has suggested that calf BIA may be more sensitive than the whole-body method [11]. We could prove a strong link between calf circumference and OH parameters, and provide further support for this emerging technique. The conventional indicators of volume overload in the non-HD population, chest X-ray or echocardiography, might not be that reliable in HD patients. Fluid oscillations associated with HD can induce organ remodeling (atrial dilatation, ventricular hypertrophy, increased pulmonary vascular resistance), and decrease the specificity and sensitivity of these techniques for fluid overload. Nevertheless, compared to BIA, conventional imaging still offers additional information that can be valuable for treatment decisions. VCD and collapsibility variations have been reported as sensitive indicators of OH, but the recommended interval of at least 1 h after HD limits the use of VC sonography in ambulatory patients [12]. Our models showed a high predictive role for VCCI in OH estimation (second best after OH_CLI_), also before HD. There are only a few studies examining the effects of HD on pulmonary functional parameters. The importance of spirometry in OH assessment has not been studied so far, and our data indicate rather an inferior role in HD.

It is evident that any of the single parameters is accurate enough to predict the extent of OH by itself. Clinical judgment of an experienced physician was the single most significant element in OH assessment, and showed the highest predictive value in combination with other variables as well. Admittedly, clinical judgment is observer-dependent and difficult to standardize. Nevertheless, the non-standardized decision choice is precisely the unique feature of clinical judgment. Studies examining different approaches to OH assessment in large patient populations typically report only the average values of the accuracy, without correlations to their standard method, which obscures the performance in individual patients. We need a method that can be applied and remains precise and reliable also in smaller groups of patients, typically encountered by dialysis physicians in routine clinical practice. Our study demonstrated that a combination of integrative clinical judgment with routine techniques is a precise and valuable tool in hydration status assessment in HD patients. BIA, a quick, reproducible and non-invasive bedside measurement, may help to identify changes in body compartments not fully appreciated by clinical or biochemical assessment. However, the most important question, whether the improved accuracy of OH assessment resulting from implementation of technological advances will also be reflected in improved patient outcomes, requires further investigation.

## Electronic supplementary material

Below is the link to the electronic supplementary material.

Supplementary material 1 (DOC 55.5 kb)
