# Supplementary material for: Clinical judgment is the most important element in overhydration assessment of chronic hemodialysis patients
Source: Clin Exp Nephrol. 2012 Nov 29;17(4):563–8. doi: 10.1007/s10157-012-0745-9 (PMC3751418; doi:10.1007/s10157-012-0745-9)
Supplement: Supplementary file 1 — Supplementary material 1 (DOC 55.5 kb) [file 10157_2012_745_MOESM1_ESM.doc]

**Electronic supplementary material (**data for online version)

We are aware of the limited statistical power of our sample and present the prognostic data as complementary information.

***Prognostic values***

In the following 9 years after initial assessment, 20 patients died (M:F, 11:9), 7 were transplanted and 3 are still on HD (M:F, 7:3). After adjustment for gender, we analyzed two arbitrary selected groups, dead (D) and alive (A) patients. Patients which remained alive were younger (D vs. A, 71.8 ± 11.9 vs. 58.8 ± 8.6; P=0.012), had higher Hb (D vs. A, 11.4 ± 1.2 vs. 12.9 ± 1.8 g/dl; P=0.01), lower post-HD PP (D vs. A, 52.0 ± 12.3 vs. 40.3 ± 14.3 mmHg; P=0.017), and a higher proportion of intracellular water ICW/TBW, which indirectly defines body cell mass (D vs. A, 45.8 ± 5.2 vs. 50.0 ± 4.5; P=0.025).

Pre-HD pulse pressure (PP, SBP-DBP), has been associated with adverse cardiovascular outcomes in HD and non-HD population. We saw a positive correlation with age (R=0.44; P=0.016) and negatively with albumin (R=-0.42; P=0.021). Serum albumin level is an important prognostic indicator. Among hospitalized patients, lower serum albumin correlates with an increased risk of morbidity and mortality. Low serum albumin can be found in chronic malnutrition that can lead to decrease of body cell mass, and was also found in our patients who died in follow-up.

| **Model** | **Adj. R²** | **AIC** | **Parameters** | **Coefficients**  **± SE** | **p** | **Predictor**  **importance** |
| --- | --- | --- | --- | --- | --- | --- |
| 1. OH_BIA_ | 0.03 | 16.5 | (Intercept)  OH_BIA_ | 2.50 ± 0.47  0.04 ± 0.11 | 0.74 | 1.0 |
| 2. ECW/BSA | 0.22 | 8.0 | (Intercept)  ECW/BSA | -1.69 ± 1.43  0.46 ± 0.15 | 0.005 | 1.0 |
| 3. OH_CLI_ | 0.35 | 2.7 | (Intercept)  OH_CLI_ | 0.79 ± 0.49  0.78 ± 0.19 | < 0.001 | 1.0 |
| * OH_CLI_ + ICW/BSA | 0.36 | 3.7 | (Intercept)  OH_CLI_ ICW/BSA | 0.06 ± 0.81  0.68 ± 0.21  0.11 ± 0.10 | 0.003  0.261 | 0.89  0.11 |
| * OH_CLI_ + ECW/BSA | 0.40 | 1.6 | (Intercept)  OH_CLI_ ECW/BSA | -1.38 ± 1.26  0.62 ± 0.20  0.27 ± 0.15 | 0.005  0.075 | 0.73  0.27 |
| 4. Parameters  (Age, pre-HD weight, pre-HD MAP, pre-HD DBP, VCCI) | 0.51 | 1.0 | (Intercept)  Age  pre-HD weight  pre-HD MAP  pre-HD DBP  VCCI | 0.32 ± 1.82  -0.03 ± 0.02 0.24 ± 0.11  -0.05 ± 0.04  0.08 ± 0.04  -0.39 ± 0.14 | 0.154  0.050  0.188  0.061  0.011 | 0.11  0.21  0.09  0.19  0.39 |
| 5. Parameters + ECW/BSA  (Age, pre-HD weight, pre-HD MAP, pre-HD DBP, VCCI) | 0.49 | 4.3 | (Intercept)  ECW/BSA  Age  pre-HD weight  pre-HD MAP  pre-HD DBP  VCCI | 0.19 ± 1.90  0.06 ± 0.18  0.02 ± 0.02 0.24 ± 0.11  -0.05 ± 0.04  0.08 ± 0.04  -0.37 ± 0.15 | 0.752  0.159  0.167  0.192  0.074  0.020 | 0.01  0.13  0.13  0.11  0.22  0.40 |
| 6. Parameters + ICW/BSA  (Age, pre-HD weight, pre-HD MAP, pre-HD DBP, VCCI) | 0.55 | -0.6 | (Intercept)  ICW/BSA  Age  pre-HD weight  pre-HD MAP  pre-HD DBP  VCCI | 0.53 ± 1.71  -0.26 ± 0.13  -0.03 ± 0.02 0.04 ± 0.01  -0.07 ± 0.04  0.13 ± 0.04  -0.26 ± 0.13 | 0.050  0.077  0.006  0.053  0.010  0.050 | 0.11  0.09  0.23  0.11  0.21  0.25 |
| 7. Parameters + OH_CLI_  (Age, pre-HD weight, pre-HD MAP, pre-HD DBP, VCCI) | 0.64 | -5.9 | (Intercept)  OH_CLI_  Age  pre-HD weight  pre-HD MAP  pre-HD DBP  VCCI | 1.49 ± 1.61  0.56 ± 0.18  0.01 ± 0.01 0.24 ± 0.11  -0.04 ± 0.03  0.07 ± 0.04  -0.31 ± 0.12 | 0.005  0.626  0.050  0.182  0.079  0.021 | 0.36  0.19  0.01  0.07  0.13  0.24 |
| * Parameters + ECW/BSA + ICW/BSA  (Age, pre-HD weight, pre-HD MAP, pre-HD DBP, VCCI) | 0.55 | 2.7 | (Intercept)  ECW/BSA  ICW/BSA  Age  pre-HD weight  pre-HD MAP  pre-HD DBP  VCCI | 0.31 ± 1.78  0.10 ± 0.17  -0.27 ± 0.13  -0.03 ± 0.02 0.04 ± 0.02  -0.08 ± 0.04  0.12 ± 0.05  -0.39 ± 0.14 | 0.555  0.050  0.077  0.024  0.052  0.012  0.011 | 0.01  0.13  0.10  0.18  0.13  0.22  0.23 |
| * Parameters + OH_CLI_ + ECW/BSA + ICW/BSA  (Age, pre-HD weight, pre-HD MAP, pre-HD DBP, VCCI) | 0.69 | -5.5 | (Intercept)  OH_CLI_  ECW/BSA  ICW/BSA  Age  pre-HD weight  pre-HD MAP  pre-HD DBP  VCCI | 1.41 ± 1.51  0.55 ± 0.17  0.12 ± 0.15  -0.26 ± 0.11  -0.03 ± 0.01 0.02 ± 0.02  -0.07 ± 0.03  0.11 ± 0.04  -0.30 ± 0.12 | 0.004  0.418  0.027  0.012  0.188  0.037  0.011  0.020 | 0.24  0.01  0.13  0.13  0.04  0.11  0.17  0.14 |
| 8. Parameters + OH_CLI_ + ECW/BSA  (Age, pre-HD weight, pre-HD MAP, pre-HD DBP, VCCI) | 0.62 | -2.5 | (Intercept)  OH_CLI_  ECW/BSA  Age  pre-HD weight  pre-HD MAP  pre-HD DBP  VCCI | 1.33 ± 1.67  0.56 ± 0.18  0.08 ± 0.16  -0.03 ± 0.01 0.02 ± 0.01  -0.04 ± 0.03  0.06 ± 0.04  -0.29 ± 0.13 | 0.006  0.633  0.039  0.893  0.183  0.098  0.040 | 0.39  0.01  0.20  0.00  0.08  0.12  0.20 |
| 9. Parameters + OH_CLI_ + ICW/BSA  (Age, pre-HD weight, pre-HD MAP, pre-HD DBP, VCCI) | 0.70 | -8.7 | (Intercept)  OH_CLI_  ICW/BSA  Age  pre-HD weight  pre-HD MAP  pre-HD DBP  VCCI | 1.66 ± 1.48  0.54 ± 0.17  -0.25 ± 0.11  -0.03 ± 0.01 0.03 ± 0.01  -0.07 ± 0.03  0.11 ± 0.04  -0.33 ± 0.11 | 0.004  0.030  0.013  0.045  0.040  0.009  0.008 | 0.22  0.11  0.15  0.07  0.10  0.17  0.18 |
